# Distinct Immunological Profiles Help in the Maintenance of Salivary Secretory IgA Production in Mild Symptoms COVID-19 Patients

**DOI:** 10.3389/fimmu.2022.890887

**Published:** 2022-05-24

**Authors:** Juliana de Melo Batista dos Santos, Jonatas Bussador do Amaral, Carolina Nunes França, Fernanda Rodrigues Monteiro, Anuska Marcelino Alvares-Saraiva, Sandra Kalil, Edison Luiz Durigon, Danielle Bruna Leal Oliveira, Silvia Sanches Rodrigues, Debora Heller, Eliane Aparecida Rosseto Welter, João Renato Rebello Pinho, Rodolfo P. Vieira, André Luis Lacerda Bachi

**Affiliations:** ^1^ Post-Graduation Program in Science of Human and Rehabilitation, Federal University of São Paulo (UNIFESP), Santos, Brazil; ^2^ ENT Research Lab, Department of Otorhinolaryngology –Head and Neck Surgery, Federal University of Sao Paulo (UNIFESP), São Paulo, Brazil; ^3^ Post-Graduation Program in Health Sciences, Santo Amaro University (UNISA), São Paulo, Brazil; ^4^ Programa de Pós-Graduação em Patologia Ambiental e Experimental, Universidade Paulista - Unip, São Paulo, Brazil; ^5^ Laboratory of Clinical and Molecular Virology, Department of Microbiology, Institute of Biomedical Science of the University of São Paulo, São Paulo, Brazil; ^6^ Scientific Platform Pasteur, University of São Paulo, São Paulo, Brazil; ^7^ Albert Einstein Institute for Teaching and Research (IIEP), Hospital Israelita Albert Einstein, São Paulo, Brazil; ^8^ Post Graduate Program in Dentistry, Universidade Cruzeiro Do Sul, São Paulo, Brazil; ^9^ Department of Periodontology, School of Dentistry, University of Texas Health Science Center at San Antonio, San Antonio, TX, United States; ^10^ Department of Gastroenterology (LIM07), Faculdade de Medicina, Universidade de São Paulo, São Paulo, Brazil; ^11^ Division of Clinical Laboratories (LIM 03), Hospital das Clínicas, Faculdade de Medicina, Universidade de São Paulo, São Paulo, Brazil; ^12^ Post-Graduation Program in Human Movement and Rehabilitation, Unievangélica, Anápolis, Brazil; ^13^ Post-Graduation Program in Bioengineering, Universidade Brasil, São Paulo, Brazil

**Keywords:** mucosal immunity, saliva, cytokines, interferon, interleukin, SARS-CoV-2

## Abstract

**Background:**

Relevant aspects regarding the SARS-CoV-2 pathogenesis and the systemic immune response to this infection have been reported. However, the mucosal immune response of the upper airways two months after SARS-CoV-2 infection in patients with mild/moderate symptoms is still not completely described. Therefore, we investigated the immune/inflammatory responses of the mucosa of the upper airways of mild/moderate symptom COVID-19 patients two months after the SARS-CoV-2 infection in comparison to a control group composed of non-COVID-19 healthy individuals.

**Methods:**

A cohort of 80 volunteers (age 37.2 ± 8.2), including non-COVID-19 healthy individuals (n=24) and COVID-19 patients (n=56) who presented mild/moderate symptoms during a COVID-19 outbreak in Brazil in November and December of 2020. Saliva samples were obtained two months after the COVID-19 diagnosis to assess the levels of SIgA by ELISA and the cytokines by multiplex analysis.

**Results:**

Salivary levels of SIgA were detected in 39 volunteers into the COVID-19 group and, unexpectedly, in 14 volunteers in the control group. Based on this observation, we distributed the volunteers of the control group into without SIgA or with SIgA sub-groups, and COVID-19 group into without SIgA or with SIgA sub-groups. Individuals with SIgA showed higher levels of IL-10, IL-17A, IFN-γ, IL-12p70, IL-13, and IFN-α than those without SIgA. In intergroup analysis, the COVID-19 groups showed higher salivary levels of IL-10, IL-13, IL-17A, and IFN-α than the control group. No statistical differences were verified in the salivary levels of IL-6 and IFN-β. Lower IL-12p70/IL-10 and IFN-γ/IL-10 ratios were found in the control group without SIgA than the control group with SIgA and the COVID-19 group with SIgA.

**Conclusion:**

We were able to present, for the first time, that associations between distinct immunological profiles can help the mucosal immunity to maintain the salivary levels of SIgA in COVID-19 patients two months after the SARS-CoV-2 infection.

## Introduction

The World Health Organization (WHO) declared, in March 2020, that humanity was facing a pandemic situation originated by the SARS-CoV-2 virus, which causes the named coronavirus disease 19 or COVID-19 (1). Since then, many studies have been conducted in order to understand not only the pathogenic aspects involved in this viral disease but also how the immune and inflammatory response is induced by this infection (2, 3).

In this respect, as the SARS-CoV-2 is a respiratory virus, and its presence in the airway elicits a local immune/inflammatory response (4) that, in a “controlled situation”, can help the host to clear the virus through the production and release of immunoglobulins, mainly secretory immunoglobulin A (SIgA) and cytokines (5–7). It is broadly accepted that the dysregulated inflammatory response promoted by the SARS-CoV-2 infection drives a cytokine storm, which is closely associated with severe symptoms and viral lethality (8). Therefore, the “dose” of immune/inflammatory responses elicited by SARS-CoV-2 infection seems to be crucial for driving a “good” or “bad” outcome in this disease. In fact, our group previously reported that individuals with severe COVID-19 presented higher levels of SIgA, interferons (IFN) type I (IFN-α and IFN-β) and type II (IFN-γ), and interleukin (IL)-37 in nasopharyngeal and oropharyngeal swabs samples as compared to the groups with mild COVID-19 and individuals with other respiratory infections (9).

In terms of immune/inflammatory responses in the airway mucosa, it has been shown that the presence of a respiratory virus, such as SARS-CoV-2, can trigger the expression of different types of cytokines that can assist not only in the clearance of the pathogenic agent, but can also generate a protective immunity against viral infection (10, 11). Moreover, it is worth mentioning that the analysis of cytokines released on airways mucosa in response to COVID-19 can be useful to define a signature of this infection, which could help to guide medical assistance, medicine development, and patients’ follow-up (12). In this sense, we have demonstrated that COVID-19 patients presenting mild symptoms showed significant correlations between different types of cytokines. One of the most interesting positive correlations found was between the levels of SIgA and IL-17A (9).

According to the literature, the production and release of cytokines by the upper airway’s mucosa, in response to respiratory infection, can elicit different immune response profiles such as Th1, Th2, and Th17, which can help in the production of mucosal specific-SIgA against the infective agent (10). Among some SIgA features, it is paramount to point out that it was reported that reduction in SIgA levels is closely associated with illness severity (13). Secretory immunoglobulin A is considered as the “first line of defense” against many different pathogens due to its capacity to directly bind and inhibit many pathogenic agents in the mucosa (14–16). Corroborating this important action, a lower level of SIgA in the airways mucosa is related to a higher risk to present upper respiratory infections (URTI) (17), particularly by a virus (18).

In terms of mucosal immunity in the upper airways, it was reported that both the oral cavity and nasal passages contain higher frequencies of sIgA+ B-cells, which suggests a similarity between these mucosal effector tissues. Based on this fact, the use of saliva is useful to evaluate not only diseases and conditions in the oral cavity, but also as well as the systemic health of individuals (19–21). These affirmations are especially valid for the immune/inflammatory response in the mucosa of the upper airways considering that saliva is an easily accessible external fluid that can be used to measure the antigen-specific SIgA antibodies following immunological challenges, such as infection and immunization, in both human and experimental animal models (14). Beyond the presence of antibodies, saliva is a biofluid composed of other biomolecules, including different types of cytokines (22, 23). Based on this information, investigation about the immune and inflammatory responses in the mucosa of the upper airway against the COVID-19 not only can improve our knowledge concerning this disease, but can allow us to better understand why some individuals are asymptomatic or why the symptomatic develop mild, moderate, or severe COVID-19. Furthermore, it is worthy to note that, until now, the overwhelming majority of studies that aimed to evaluate the antibodies and cytokines profile in COVID-19 patients were focused on systemic aspects, mainly in patients with severe disease, whom, in general, presented the cytokine storm. Therefore, in the present study, we evaluated the immune/inflammatory responses in the mucosa of the upper airway, particularly assessing SIgA and cytokines, two months after SARS-CoV-2 infection in a group of patients who presented mild/moderate symptoms in comparison to a control group composed of healthy individuals.

## Materials and Methods

### Subjects of the Study

In the present study, 80 individuals (mean age 37.2 ± 8.2), 28 men and 52 women, were enrolled. The volunteers were distributed into two groups: the control group (n=24) was composed of non-COVID-19 healthy individuals and the COVID-19 group (n=56) was composed of individuals infected with SARS-CoV-2, who presented mild/moderate symptoms between the months of November and December of 2020, during a COVID-19 outbreak in Brazil. We clarify that on this occasion none of the volunteers were submitted to the vaccination for COVID-19. All the participants were informed about the study and signed the informed consent form previously approved by the Ethics Committee of the Albert Einstein Hospital (number 4.159.565). It is noteworthy to highlight that both the study and all experiments were performed in accordance with the Declaration of Helsinki. Data concerning age, gender, and clinical parameters, including the COVID-19 symptoms, are shown in **Table 1**.

**Table 1:** Demographic, clinical characteristics, and symptoms presented by the patients who composed the COVID-19 sub-groups without or with SIgA.

Parameter	CONTROL		COVID-19		*p* value
	Without SIgA (n=10)	With SIgA (n=14)	Without SIgA (n=17)	With SIgA (n=39)	
Age	37.7 ± 9.5	40.5 ± 11.4	35.5 ± 4.8	35.4 ± 7.4	
Male	6	4	6	12	> 0.05
Female	4	10	11	27	> 0.05
M/F ratio	1:0.66	1:2.5	1:1.84	1:2.25	> 0.05
***Clinical characteristics***			***n***	***n***	
Comorbidity			11	18	> 0.05
Hypertension			1	5	> 0.05
Obesity			0	3	> 0.05
Asthma			2	1	> 0.05
Pregnancy			0	1	> 0.05
Smoking			0	2	> 0.05
Presence of COVID-19 symptoms		10	16	> 0.05
Fever			5	4	> 0.05
Cough			4	9	> 0.05
Dyspnea			3	6	> 0.05
Anosmia			6	12	> 0.05
Ageusia			4	11	> 0.05
Sore throat			3	7	> 0.05
Myalgia			6	10	> 0.05
Chills			4	8	> 0.05
Coryza			5	9	> 0.05
Headache			7	13	> 0.05
Nausea/Vomiting			2	5	> 0.05
Diarrhea			3	9	> 0.05
Fatigue			6	10	> 0.05
Mental confusion			2	0	> 0.05
Chest pain			3	4	> 0.05
Days of symptoms			28 +/- 43	22+/-27	> 0.05
Days of symptoms (range)			1 - 144	08 - 120	

n, Number of Volunteers presenting each clinical characteristic.

### Determination of Virus Infection by RT-PCR

The diagnosis for SARS-Cov-2 infection was carried out by real-time (RT) PCR using nasal/oropharyngeal samples obtained in the individuals of the COVID-19 group and healthy individuals. The RT-PCR kits XGEN MASTER COVID-19 (Mobius, Pinhais, Paraná, Brazil); COBAS^®^ SARS-CoV-2 Test (Roche Molecular Systems, Branchburg, NJ, USA); Xpert^®^Xpress SARS-CoV-2 (Cepheid, Sunnyvale, CA, USA); and Abbott RealTime SARS-C0V-2 (Abbott Molecular Inc.,DesPlaines, IL,USA) were used following manufacturer’s instructions (24–32).

### Saliva Sampling

Unstimulated saliva was self-collected by the COVID-19 patients or healthy non-COVID-19 volunteers in a sterile 50-mL tube as previously described (33) in the “Hospital Israelita Albert Einstein” located in São Paulo, Brazil. Regarding the COVID-19 group, the samples were obtained 55 to 60 days after the COVID-19 diagnosis. In relation to the healthy non-COVID-19 volunteer group, none of them presented SARS-CoV-2 infection on the salivary sample collection day. All saliva samples were centrifuged at 3000 rpm for 5 min and the supernatants were stored in -80°C to perform cytokines and secretory immunoglobulin A assays. The volunteers enrolled in this study presented good oral health conditions at the moment of salivary samples collection. In addition, it is noteworthy to point out that all the volunteers were submitted for clinical examination in order to attest to their good health status before the saliva sampling.

### Determination of Secretory Immunoglobulin A (SIgA)

Secretory IgA immunoglobulin (SIgA) was detected by ELISA in-house test, which was previously standardized, in order to define not only the optimum concentration of SARS-CoV-2 antigens but also the better saliva samples dilution. Briefly, 96-well plates (Corning, New York, USA) were coated with a mixture of antigens (0.12 ug/mL in sodium carbonate–sodium bicarbonate buffer) from nCoV-PS-Ag7 (Fapon Biotech Inc., Dongguan, China) containing the nucleoprotein (N), membrane (M) and spike (S) and was incubated overnight. Unspecific binding of antibodies was avoided by blocking with the buffer PBS-BSA-T containing 1% of bovine serum albumin (Invitrogen by Thermo Fisher Scientific, Vienna, Austria) in PBS (1X, pH: 7.3) + 0.05% of Tween 20 (Synth, Diadema, Brazil) at 37°C for 2h. After washing three times with a PBS-T solution (PBS 1X, pH:7.3 + 0.05% of Tween), 100μL of saliva [diluted at 1:2,000 in PBS-BSA (PBS 1X, pH: 7.3 + 0.1% of BSA)] was added and incubated for 2h at 37°C. After washing three times with a PBS-T solution, it was added the secondary antibody conjugated with horseradish peroxidase diluted at 1:2,000 (in PBS-BSA) of goat anti-human IgA (Sigma-Aldrich Co., Deisenhofen, Germany). After incubation for 1h at 37°C and three PBS-T washes, 100 μL of TMB solution (3.3′.5.5′- tetramethylbenzidine. Thermo Scientific, Massachusetts, USA) was added and incubated for 10 min at room temperature, avoiding direct exposure to light. The reaction was stopped by adding a solution of sulfuric acid (0.2 N) to each well, and the optical density at 450nm was measured.

### Determination of Cytokines

Cytokine concentrations were determined in the saliva samples by a multiplex assay (LEGENDplex™ bead-based multiplex assays, Biolegend, San Diego, CA, USA). The biomarkers assessed were: IL-6, IL-10, IL-12p70, IL-13, IL-17A, IFN-α, IFN-β, and IFN-γ following the manufacturer’s instructions. In this regard, all salivary samples were initially diluted 2-fold and after 25microL of the sample diluted was used to perform this assay. The concentration of cytokines was calculated using appropriate standard curves (following instructions from manufacturers). The linearity of the multiplex assay was within the 2.4–10,000pg/mL range, which includes the range of sample determinations. The correlation coefficients of all standard curves were ranged from 0.95 to 0.99, while intra-assay variance coefficients were 3–5%, and interassay variance coefficients were 8–10%. Analysis was performed with the BD Accuri™ C6 Plus Flow Cytometer (BD Biosciences San Jose. CA. USA) and the data obtained were analyzed with LEGENDPlex™ V8.0 software (Biolegend).

### Statistical Analysis

All data obtained from the SIgA and cytokines analysis were initially compared with the Gauss curve and the normality for each parameter assessed was determined by the Shapiro-Wilk test, followed by the homogeneity of variance analysis by the Levene test. Salivary concentrations of SIgA and the cytokines in the volunteer groups were analyzed using the Mann-Whitney test and were presented as the median with the respective quartiles. In addition, the correlation test was performed by Spearman’s test. Differences between age, number of men and women, and the salivary concentrations ratio between IL-10 and the other cytokines were analyzed using the Student T-test and were presented as mean and standard deviation, whereas the differences between the clinical characteristics and symptoms were evaluated using the Chi-square test. Significance was established with α risk at 5.0% level (p ≤ 0.05) and all the analysis was performed data using GraphPad Prism (version 8.1.2) software.

## Results

**Figure 1:**
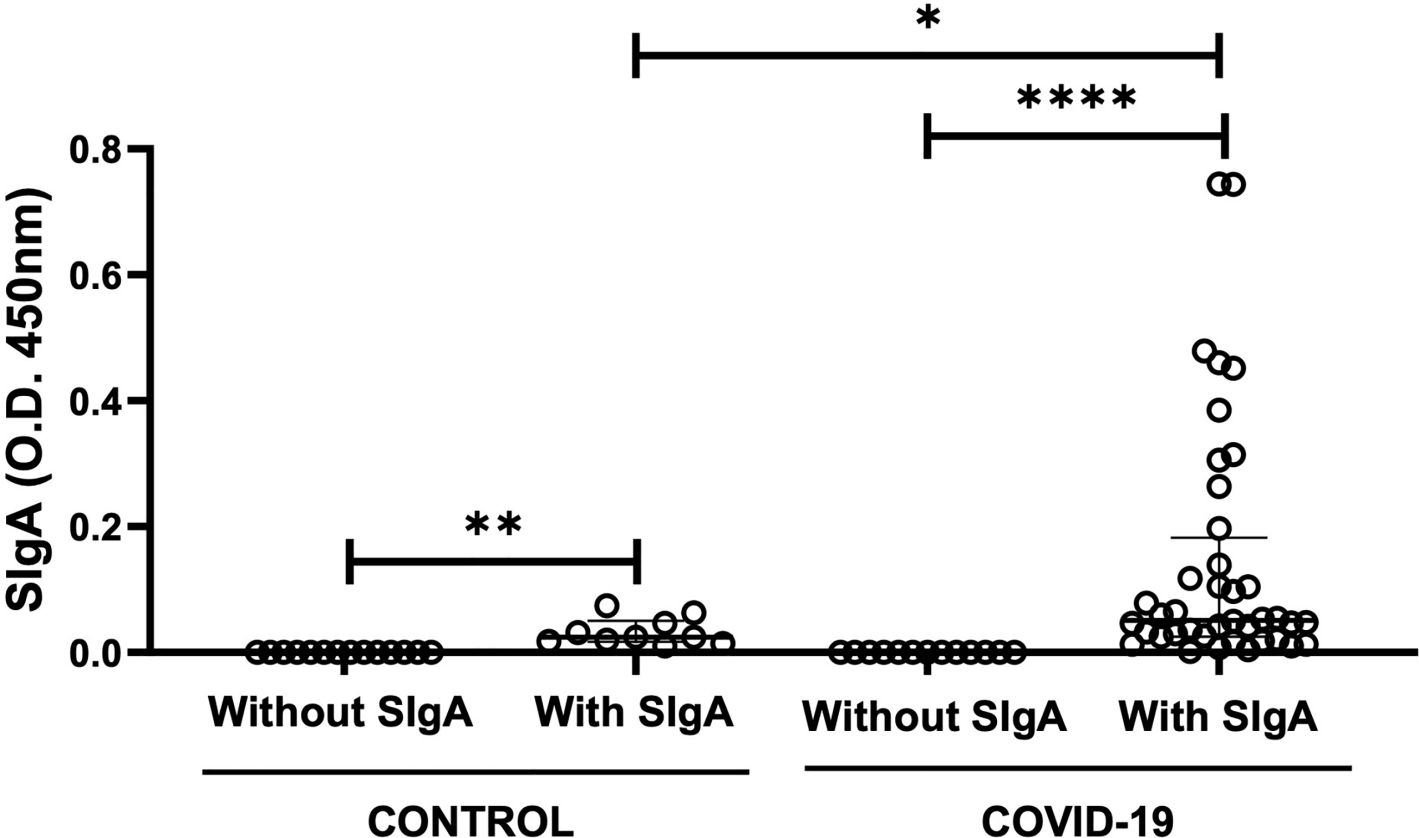
Comparison of the levels of salivary specific-SIgA for SARS-CoV-2 between control and COVID-19 groups with or without the presence of SIgA. The level of significance was established at 5% (*p < 0.05; **p < 0.05; ****p < 0.0001).

As shown in **Figure 1**, some volunteers in the control group did not present SIgA for SARS-Cov-2 antigens (n=10) as expected, whereas other volunteers presented this antibody in saliva (n=14). It is worthy to note that, as mentioned in the “Material and Methods” section, these healthy volunteers were allocated to the control group based on the observation that all of them were RT-PCR negative for COVID-19. In addition, the volunteers infected by SARS-Cov-2 were also separated into two groups in accordance with the presence of SIgA: COVID-19 with SIgA (n=39) and COVID-19 without SIgA (n=17). Significant differences were found in the intragroup analysis between the volunteers in the subgroups control (*p* = 0.002) or COVID-19 (*p* < 0.0001). Moreover, in the intergroup analysis, the salivary SIgA levels observed in the COVID-19 group were higher than the levels found in the control group (*p* = 0.04).

Since the groups were distributed due to the presence or absence of SIgA in saliva, **Table 1** presents the number of individuals allocated in the control group without SIgA or with SIgA, as well as in the COVID-19 group without SIgA or with SIgA. In addition, it presents the age, the number of men and women, and its ratio in each volunteer group along with the comorbidities and symptoms found in COVID-19 groups during the SARS-CoV-2 infection. In a general way, no differences were observed in these parameters.

**Figure 2:**
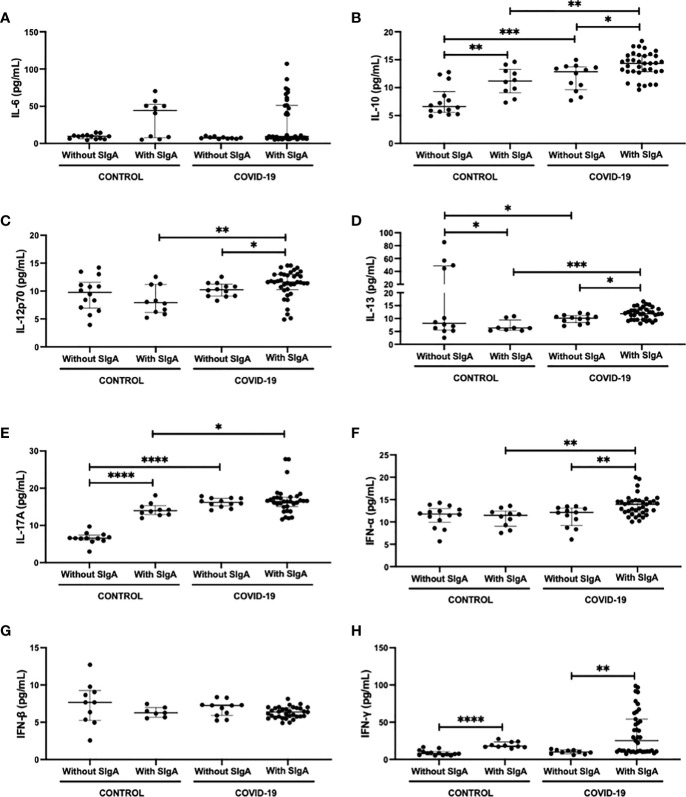
Comparison of the salivary cytokines levels of IL-6 **(A)**, IL-10 **(B)**, IL-12p70 **(C)**, IL-13 **(D)**, IL-17 **(E)**, IFN-α **(F)**, IFN-β **(G)**, and IFN-γ **(H)** between control without or with SIgA and COVID-19 without or with SIgA group. Values are presented in the median and interquartile range. Statistical analysis: Mann-Whitney test. The level of significance was established at 5% (*p < 0.05; **p < 0.01; ***p < 0.001; ****p < 0.0001).

**Figure 2** shows the analysis of salivary cytokines, both pro (IL-6, IL-12p70, IL-13, IL-17A, IFN-α, IFN-β, and IFN-γ) and anti-inflammatory (IL-10), in the volunteers enrolled in the present study. Concerning the results obtained in the intragroup evaluation, it was found that the control group with SIgA presented higher levels of IL-10 (**Figure 2B**), IL-17A (**Figure 2E**), and IFN-γ (**Figure 2H**), as well as lower levels of IL-13 (**Figure 2D**) than the values observed in the control group without SIgA. In a similar way, the COVID-19 group with SIgA presented higher levels of IL-10 (**Figure 2B**) and IFN-γ (**Figure 2H**) than the values found in the COVID-19 group without SIgA. However, increased salivary levels of IL-12p70 (**Figure 2C**), IL-13 (**Figure 2D**), and IFN-α (**Figure 2F**), and no differences in IL-17 levels (**Figure 2E**) were also observed in the COVID-19 group with SIgA as compared to the results obtained in the COVID-19 group without SIgA. In addition to these findings, it was observed in the intergroup analysis that the COVID-19 group without SIgA showed higher levels of IL-10 (**Figure 2B**), IL-13 (**Figure 2D**), and IL-17A (**Figure 2E**) than the control group without SIgA. In relation to the groups with SIgA, increased levels of IL-10 (**Figure 2B**), IL-13 (**Figure 2D**), IL-17A (**Figure 2E**), and IFN-α (**Figure 2F**) were found in the COVID-19 group as compared to the values observed in the control group. No statistical differences were verified in the salivary levels of IL-6 (**Figure 2A**) and IFN-β (**Figure 2G**).

**Table 2** presents the results obtained in the Spearman coefficient correlation analysis. Interestingly, a positive correlation between IFN-α and IL-12p70 was verified in all volunteer groups. In a different way, whereas IFN-α showed a positive correlation with IL-6 in the control group without SIgA, the IFN-α showed a positive correlation with IL-10 in the control group with SIgA, with IFN-γ in the COVID-19 group with SIgA, and with IL-13 in both COVID-19 groups. Concerning IL-6, it was found a positive correlation with IL-12p70 in the control group without SIgA, whereas the control group with SIgA IL-6 showed negative correlations with IL-13 and IFN-γ. Particularly in the COVID-19 groups, IL-6 presented a positive correlation with IFN-γ and also IL-12p70 presented a positive correlation with IL-17A. In addition, the groups that presented SIgA showed a positive correlation between IL-12p70 and IL-10. Another cytokine that showed significant correlations in the groups presenting SIgA was IL-13, but with different cytokines, since in the control group with SIgA, a negative correlation with IFN-β was observed, and in the COVID-19 group with SIgA, two other positive correlations were evidenced, with IFN-γ and IL-17A. Lastly, the COVID-19 group without SIgA exclusively showed a positive correlation between IL-12p70 and IL-13, whereas the COVID-19 group with SIgA presented positive correlations between the levels of SIgA and IFN-γ, as well as IL-17A.

**Table 2:** Significant correlations between analysis of SIgA and cytokines of COVID-19 and control with and without SIgA groups.

Group	Correlation		Pearson r	*p* value
Control without SIgA	IL-6	IL-12p70	0.626	0.019
	IL-6	IFN-α	0.596	0.027
	IL-12p70	IFN-α	0.824	0.001
Control with SIgA	IL-6	IL-13	-0.661	0.044
	IL-6	IFN-γ	-0.818	0.006
	IL-10	IL-12p70	0.721	0.023
	IL-10	IFN-α	0.794	0.009
	IL-12p70	IFN-α	0.879	0.002
	IL-13	IFN-β	-0.721	0.023
COVID-19 without SIgA	IL-6	IFN-γ	0.841	0.001
	IL-12p70	IL-13	0.812	0.001
	IL-12p70	IL-17A	0.628	0.023
	IL-12p70	IFN-α	0.749	0.004
	IL-13	IFN-α	0.575	0.043
COVID-19 with SIgA	SIgA	IL-17A	0.427	0.001
	SIgA	IFN-γ	0.334	0.025
	IL-6	IFN-γ	0.560	0.001
	IL-10	IL-12p70	0.555	0.001
	IL-12p70	IL-17A	0.334	0.035
	IL-12p70	IFN-α	0.537	0.001
	IL-13	IL-17A	0.335	0.034
	IL-13	IFN-α	0.543	0.001
	IL-13	IFN-γ	0.420	0.007
	IFN-α	IFN-γ	0.342	0.027

**Figure 3:**
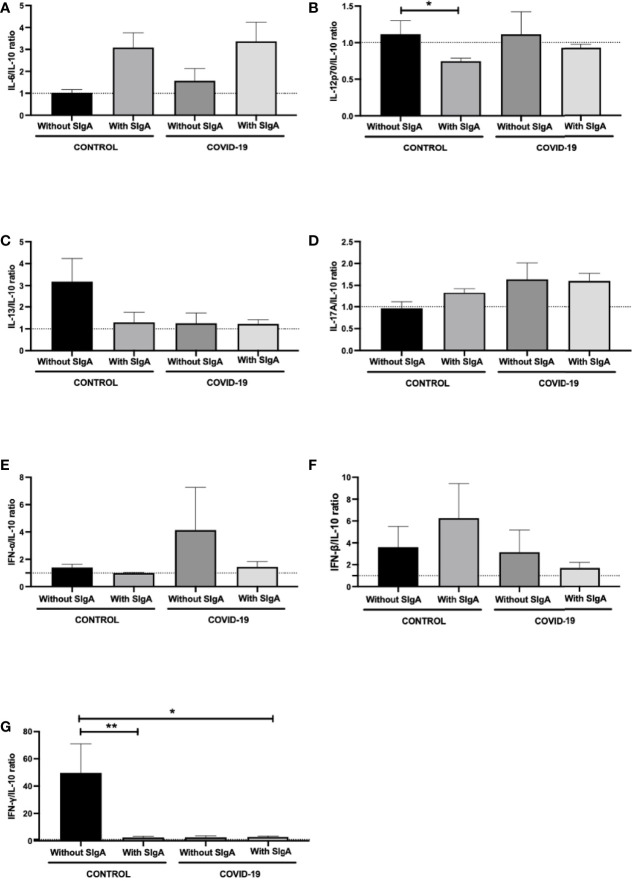
Comparison of the salivary cytokines ratios of IL-6/IL-10 **(A)**, IL-12p70/IL-10 **(B)**, IL-13/IL-10 **(C)**, IL-17/IL-10 **(D)**, IFN-α/IL-10 **(E)**, IFN-β/IL-10 **(F)**, and IFN-γ/IL-10 **(G)** between control without or with SIgA and COVID-19 without or with SIgA group. Values are presented in the median and interquartile range. Statistical analysis: Kruskal Wallis test. The level of significance was established at 5% (*p < 0.05; **p < 0.01).

**Figure 3** shows the analysis of the ratio between salivary levels of IL-10 and the other pro-inflammatory cytokines assessed in this study. It was found a significant decrease in the IL-12p70/IL-10 ratio (**Figure 3B**) between the control group without SIgA and the control group with SIgA, as well as in the IFN-γ/IL-10 ratio (**Figure 3G**) between the control group without SIgA, the control group with SIgA, and the COVID-19 group with SIgA. No other differences were observed.

**Figure 4:**
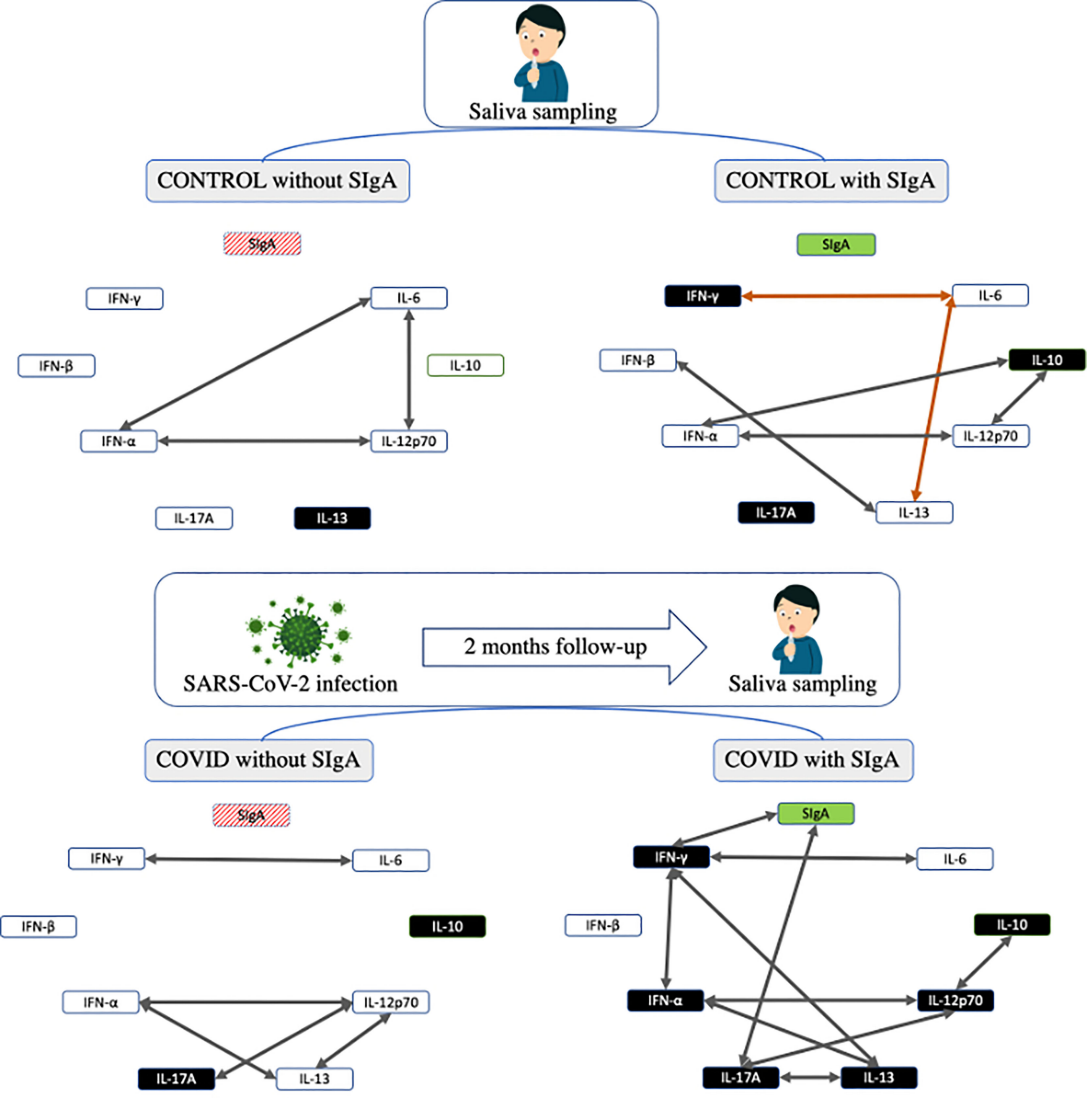
Summarized representation of the main results found in the study. The boxes representing the cytokines are colored in black color indicating an increase in their concentration when compared to the other groups. The arrows represent the correlations found in the study, in which positive correlations appear in gray and negative in orange.

**Figure 4** summarizes all data found in the volunteer groups of this study.

## Discussion

The present study shows, for the first time, that two months after SARS-CoV-2 infection, the COVID-19 group with SIgA showed increased salivary SIgA levels than the control group with SIgA, as well as pro-inflammatory cytokines, such as IL-12p70, IL-13, IL-17A, IFN-α, and IFN-γ, and the anti-inflammatory cytokine IL-10, as compared to the other groups. In an interesting way, no differences in the salivary levels of IL-6 and IFN-β were verified between the groups. Furthermore, the correlation analysis demonstrated remarkable associations in all volunteer groups, highlighting the results obtained in the COVID-19 group with SIgA.

Corroborating to the present study, our group (9) has previously demonstrated a significant positive correlation between IL-17A and SIgA in COVID-19 patients presenting mild/moderate symptoms. According to the literature, IL-17A is one of the major cytokines involved in the Th17 immune response, including the upper airway mucosal immunity, by improving its protection in association with a specific-SIgA response (10). Hence, it was reported that IL-17 can enhance the expression of the polymeric immunoglobulin receptor (pIgR), which is responsible to mediate the transport of polymeric immunoglobulins such as SIgA, across the mucosal epithelial cells, delivering this antibody to the mucosal surface (14). In fact, it is utmost of importance to highlight that IL-17 is associated with an upper airway mucosal protective immunity since its neutralization impaired the immune response against not only nasal vaccination but also influenza infection (10, 34, 35). In a recent review, Hoffmann et al. (2021) (36) showcased the corollary action of IL-17 in the airway’s viral infection and secondary bacterial infections (37). In this context, in association with IL-22, IL-17 can also regulate the function of the epithelial barrier and mediate host response to infections (38–40) by inducing the production of antimicrobial proteins and mucus, recruitment of monocytes and neutrophils, favoring tight junction formation, and the mucosal repair (41–44). Moreover, it has been reported that the improvement of secondary bacterial infections clearance during influenza infection was related to IL-17 production (45). In line with these findings, Smith and collaborators (14) declared that “the strategic positioning of TH17 cells at barriers surfaces reflects their importance in the neutralization of pathogens”. So, as COVID-19 is also a respiratory disease, the production of IL-17A in the upper airway of individuals with SARS-Cov-2 infection can be crucial to elicit an immune response that limits disease severity (36), particularly by inducing specific-SIgA for COVID-19 as observed in the present study.

Based on the previous report of Mahallawia and collaborators (2018) (44), in which it was shown that patients with acute MERS-CoV infection presented both Th1 and Th17 responses, our findings show a significant positive correlation between IL-12p70, a classical Th1 cytokine, and IL-17 in the COVID-19 groups. Beyond that, higher levels of IL-12p70 and IL-17 were also observed in the COVID-19 group with SIgA compared to the COVID-19 group without SIgA. Therefore, it shows that the SARS-CoV-2 infection can elicit a similar immune response denoted by MERS-CoV. In terms of mucosal immunity, IL-12p70 is a cytokine that not only can induce interferon production (46), but can also act directly in B cells (47) stimulating their growth and immunoglobulin secretion (48, 49). Moreover, it was demonstrated that IL-12 can be important to enhance the pIgR expression on upper airways (50) and also that the nasal immunization with this cytokine was able to increase antigen-specific antibody response (14). Taking these data together, we can hypothesize that the concomitant increase of IL-12p70 with IL-17A observed in the COVID-19 group with SIgA could be an important feature in order to promote an efficient stimulation of the immune response in the airway’s mucosa, leading to SIgA production. However, this association between the salivary levels of IL-12p70 and IL-17A, seemingly, is not the unique factor able to induce the SIgA production in COVID-19 patients, since the COVID-19 group without SIgA did also present the same correlation.

In this sense, another interesting finding in the samples collected after two months of SARS-CoV-2 infection in the COVID-19 group with SIgA was not only the increased IL-13 levels but also the positive correlation between IL-13 and IL-17. Although elevations in the plasma levels of both IL-13 and IL-17, among other cytokines, were observed in symptomatic patients infected by SARS-CoV-2 as compared to controls (51), higher IL-13 levels in saliva of COVID-19 patients presenting SIgA than COVID-19 patients without SIgA or control with SIgA has not been reported before. It is broadly known that IL-13 is a cytokine involved in the Th2 immune response and its elevation is closely associated with several respiratory diseases, such as asthma, COPD, polyposis, and allergy, notably involved in hypersecretion of mucus in such diseases. However, it is also accepted that some Th2-derived cytokines, including IL-13, are able not only to induce IgA+ B cells proliferation, but also the differentiation of these cells into IgA-secreting plasma cells (52), which can promote the increase of SIgA production in the mucosa.

In terms of viral infection, it was demonstrated that the presence of innate lymphocyte cells (ILC), a type of immune cell that shares many characteristics of the CD4+ T helper cell but without adaptive immunity receptors/lymphoid lineage expression, can induce the production of IL-13 (53, 54) when it presents the Th2 profile (called ILC2) in the respiratory tract. Concerning ILC2 cells, the number of these cells increased in the mucosa. particularly in the intestines, lungs, and tonsils (55), playing a prominent role in the maintenance of the epithelial cell barrier since its depletion led to profound damages in the epithelial barrier following H1N1 infection (56). Also, it was demonstrated that in an H1N1 infection there was an accumulation of ILC2 cells in the lung, regardless of viral load (57). Regarding ILC cells profiles, as previously mentioned, ILC2 cells were associated with a Th2 immune profile characterized by the production of IL-13, whereas type 1 ILC (ILC1) cells produce interferon-gamma (IFN-γ), type 3 ILC (ILC3) cells IL-17A, and IL-22 (58). However, recently it was reported that ILC2 cells also can transdifferentiate to an ILC3-like cell and produce IL-17, which shows that the ILC2 cells in the airways mucosa present remarkable plasticity and can contribute with ILC3 cells in IL-17 production following infection. Therefore, the positive correlation between IL-13 and IL-17 could be putatively attributed to ILC2 cells responses, which lead to the improvement of the mucosa protective immune response, particularly by eliciting the SIgA production.

Corroborating this suggestion that the association between IL-13 and IL-17 can improve the conditions to promote SIgA production in COVID-19 patients, including two months after SARS-CoV-2 infection, we found that the COVID-19 group without SIgA did not show this same association. Instead, the COVID-19 group without SIgA showed an exclusive positive correlation between the salivary levels of IL-13 and IL-12p70.

According to the classical immunological knowledge, both in physiologic or even some infection and inflammation situations, when the immune response is driven to the Th1 profile (IL-12), the Th2 profile (IL-13) is diminished, or even some cytokines belonging to the same Th response can be preferentially produced than others (59, 60). For instance. it was demonstrated that during RSV responses, the IL-12 production was negatively related to IL-13 production, that IL-12/IL-13 axis was central to elicit an effective or innefective immune response to RSV infection and central to dictate the infection severity (61). Furthermore, it has been pointed out that the control of IL-13 release is crucial to avoid or minimize the dangerous effect of this cytokine in some diseases and infection processes in the airways (50, 61–63) and the positive correlation between the levels of IL-13 and IL-12p70 could represent a remarkable way to putatively control the harmful IL-13 effects in COVID-19 patients. Therefore, this association also could be responsible for the lack of association between IL-13 and IL-17, two months after SARS-CoV-2 infection, which led some COVID-19 patients to not present salivary SIgA levels.

In an interesting finding two months after SARS-CoV-2 infection, COVID-19 group patients with SIgA, the salivary level of IL-13 did not show a positive correlation with IL-17 and IFN-γ. This last association can favor the activation of an effective immune response to SARS-CoV-2 infection since it was reported that the concomitant production of IFN-γ and IL-13 in the airways increased the numbers of CD11c-positive cells expressing MHC class II, as well as the co-stimulator molecule CD86 (64), which can improve not only antigen-presentation but also the activation of CD4+ T cell. Furthermore, Jartti and collaborators (2014) (65) demonstrated that the presence of viral infection on tonsils or nasopharyngeal mucosa induced an “unexpected” strong correlation between IL-13 and the cluster of antiviral cytokines, which included all types of interferons.

According to these authors, the concomitant secretion of IL-13 and antiviral interferons can decrease inflammation and injury due to the capacity of IL-13 in inhibiting the pro-inflammatory factors synthesis by monocytes and macrophages during viral infection (66). Based on the information presented above, we can putatively suggest that, the positive correlation between IL-13 and IFN-α observed in both COVID-19 groups two months after the SARS-CoV-2 infection was important to control the inflammation and consequently minimize an eventual tissue injury in the airways, and, also, the positive correlation between IL-13 and IFN-γ can help the activation of the immune response leading to the SIgA production.

Beyond these observations, the COVID-19 group with SIgA showed increased IFN-α and IFN-γ levels as compared to the COVID-19 group without SIgA, as well as a positive correlation between these interferons. Higher IFN-α and IFN-γ levels were verified in patients with MERS-CoV (67) and SARS-CoV (9, 68), and it was postulated that these elevations could be useful both to improve the antigen presentation and also to develop a robust antiviral response against these infections (44, 69).

Although the elevation of IFN-γ in COVID-19 patients with mild/moderate symptoms have already been demonstrated by our group (9), the present study found significant positive correlations between the levels of IFN-γ with SIgA, IFN-α, IL-6, and IL-13 which were found in the same group in salivary samples obtained two months after SARS-CoV-2 infection, which we found particularly concerning.

It is noteworthy to point out that regardless of the type of interferons [type-I (IFN-α and IFN-β); type II (IFN-γ); or type-III (IFN-λ)], in a viral infection, these molecules are the main antiviral cytokines that promote the infection control and the ability to elicit an adaptive immune response to improve the viral clearance (70). Regarding the coronavirus infection, mainly in MERS-CoV and SARS-CoV infections, it was documented that the elevation in interferons both type-I and type-II was able to elicit an efficient host defense through its essential property of inhibiting coronavirus replication (9, 70). In terms of SARS-CoV-2 infection, the presence of the protective action of IFNs in the upper airway is very important to promote viral clearance with or without mild/moderate symptoms (71). Therefore, the increase in salivary levels of IFN-α and IFN-γ, especially in the COVID-19 group with SIgA, may elicit an effective immune response in the mucosa of the upper airway leading to SIgA production.

In this respect, according to the literature, IFN-γ is another cytokine that, similar to IL-17, is capable to upregulate the pIgR expression in the mucosal epithelial cells, which can drive to the better secretion of SIgA in the mucosa surfaces. Furthermore, it was reported that, in response to pro-inflammatory stimulus, IFN-γ can also up-regulate the expression of MHC class II molecules on mucosal epithelial cells. It is also paramount to highlight that the elevation of the IFN-γ levels concomitant with IL-6 by cells in the submandibular glands was able to create conditions for IgA synthesis in this site (14).

Thus, its direct effect on MHC class II and pIgR expression on the mucosal epithelial cells and the positive correlation with IL-6, demonstrates that IFN-γ is a very important cytokine to induce a favorable molecular environment in the mucosa of the upper airway to promote the secretion of SIgA (14). However, our finding that the COVID-19 group without SIgA also presented a positive correlation between IFN-γ and IL-6, two months after the SARS-CoV-2 infection, can reinforce the importance of the other features formerly described in the response necessary to guarantee SIgA production.

Interestingly, in the control group with SIgA, negative correlations were observed between the levels of IFN-γ and IL-6, between IFN-β and IL-13, and between IL-13 and IL-6. These findings are in agreement with the classical notion that, in a physiologic situation, without infection and inflammation, when the immune response is driven to the Th1 profile (IFN-γ), the Th2 profile (IL-6 and IL-13) is diminished. However, Th1 and Th2 cytokines may co-exist depending of the stimuli. Therefore, we can suggest that in this asymptomatic group, the presence of SARS-CoV-2 infection led to the activation of the immune response in the mucosa of the upper airway and after the viral clearance, confirmed by the PCR test negative, the physiological conditions have been stabilized.

Another very interesting result is related to the positive association between the salivary levels of IFN-α and IL-12p70, regardless of the SARS-Cov-2 infection or SIgA production. In agreement with the literature, both IFN-α and IL-12 are cytokines present in the The immune response and can induce the IFN-γ production in the oral cavity (72, 73). It was reported that low doses of IFN-α are able to prime the mucosal immune responses and that its functions are upstream of IL-12 action in the Th1 response against different antigens (self and non-self) and pathogens, mainly viruses in the oral cavity (74, 75). Regarding IFN-α action, its presence can induce the differentiation of monocytes to dendritic cells, when released during antigen presentation to CD4 T lymphocytes. This not only elicits signals in favor of Th1 immune response but also promotes upregulation of high-affinity IL-12 receptors (IL-12b1/b2), which leads the Th1 cells to be more responsive to IL-12 actions, as well as be useful to amplify the CD8 T lymphocyte resistance to viral infections (74, 76, 77). Furthermore, IFN-α also demonstrated a potential capacity to improve the neutrophils’ antifungal activity in the oral cavity (78, 79). Taking together all these data, our observation of the positive correlation between IFN-α and IL-12p70 could be understood as a hallmark of oral cavity immunity since all the volunteer groups presented this same association.

Specifically in the control group without SIgA, the positive correlation between IL-6 and IFN-α or IL-12p70 showed an interesting regulation of the immune response in the oral cavity since as formerly described, IL-6 is associated with Th2 immune response whereas IFN-α and IL-12 are associated with Th1 immune response. These positive correlations can corroborate our previous report that this association (Th1/Th2) is favorable to create a mucosal environment more protective by guaranteeing conditions to produce SIgA (14), particularly in response to the presence of a pathogenic agent. Furthermore, IL-6 is a cytokine that presents both pro and anti-inflammatory effects, depending on the context. It is accepted that this cytokine can be produced by a variety of cells, such as neutrophils, macrophages, fibroblasts, keratinocytes, endothelial cells, and also that it is involved in several biological processes, such as antibody production, T and B cells activation and differentiation, hematopoiesis, vascular permeability, and angiogenesis (80–82). In the oral cavity, IL-6 is a crucial cytokine involved in the host response against bacterial infection (83) and together with IFN-α, is important in host defense to influenza infection (84). So, the association between these cytokines allows us to speculate that this immune profile is necessary to create a protective environment improving the host defenses against potential pathogens.

Alternatively to what is described above, the groups that presented SIgA in saliva showed a positive correlation between IL-10 and IFN-α and/or IL-12p70, which demonstrates that the control of inflammation was elicited. It is broadly known that IL-10 is one of the most important anti-inflammatory cytokines and its production is crucial to regulate the inflammatory process (85). In the present study, we show that salivary IL-10 levels were increased in the COVID-19 groups (two months after the SARS-CoV-2 infection) and also in the control group that presented SIgA, demonstrating that IL-10 production was promoted and necessary to control the inflammation induced by SARS-CoV-2 infection. To support this idea, we observed no difference in the analysis of the ratio between salivary levels of IL-10 and the other pro-inflammatory cytokines, with the exception of a significant decrease of IL-12p70/IL-10 and IFN-γ/IL-10 ratios, which were closely associated with the increase of IL-10. According to the literature, the analysis of the ratio between pro and anti-inflammatory cytokines is considered a keystone that can clarify whether the inflammatory process is controlled (86), and mitigating dangerous situations that occur when the inflammation is not regulated, such as the cytokine storm in SARS-CoV-2 infection (87). Therefore, we can putatively suggest that IL-10 was able to control the inflammation in the COVID-19 groups, even two months after the SARS-CoV-2 infection, and in the control group with SIgA, which allows us to hypothesize that this regulation contributed to these volunteers being asymptomatic or presenting moderate/mild symptoms.

Taking our findings together, we were able to reinforce the importance of IL-17 in the immune response of airways’ mucosa and show, for the first time, relevant characteristics and associations between cytokines in COVID-19 groups, two months after the SARS-CoV-2 infection, in producers or non-producers of salivary specific-SIgA for SARS-CoV-2 virus.

## Data Availability Statement

The raw data supporting the conclusions of this article will be made available by the authors, without undue reservation.

## Ethics Statement

The studies involving human participants were reviewed and approved by Ethics Committee of the Albert Einstein Hospital. The patients/participants provided their written informed consent to participate in this study.

## Author Contributions

JS, RV, and AB conceived the study. JS, JP, RV, ED, DO, and AB participated in the planning and development of this study. JS, JA, CF, FM, AA-S, SK, and EW participated in the laboratory analysis. SR, DH, EW, and JP laboratory diagnosis analysis, hospital clinical evaluation, sample collection, and patients’ clinical evaluation. JS, RV, and AB wrote the initial and final draft of the manuscript. All authors contributed to the article, revised the final draft, and approved the submitted version of the manuscript.

## Funding

Funding was granted by Fundação de Amparo à Pesquisa do Estado de São Paulo (FAPESP), Grants No. 2016/20045-7 and 2020/06409-1 (ED), No. 2012/15165-2 (RV) and 2019/14115-0 (AB).

## Conflict of Interest

The authors declare that the research was conducted in the absence of any commercial or financial relationships that could be construed as a potential conflict of interest.

## Publisher’s Note

All claims expressed in this article are solely those of the authors and do not necessarily represent those of their affiliated organizations, or those of the publisher, the editors and the reviewers. Any product that may be evaluated in this article, or claim that may be made by its manufacturer, is not guaranteed or endorsed by the publisher.
