# Supplementary material for: Distinct Immunological Profiles Help in the Maintenance of Salivary Secretory IgA Production in Mild Symptoms COVID-19 Patients
Source: Front Immunol. 2022 May 24;13:890887. doi: 10.3389/fimmu.2022.890887 (PMC9171398; doi:10.3389/fimmu.2022.890887)
Supplement: Supplementary file 1 [file Table_1.docx]

# Supplementary Material

**Table S1:** Correlations between analysis of SIgA and cytokines of COVID-19 and control with and without SIgA groups.

| Groups | Parameters | SIgA | IL-6 | IL-10 | IL-12 | IL-13 | IL-17A | IFN-ɑ | IFN-β | IFN-γ |
| --- | --- | --- | --- | --- | --- | --- | --- | --- | --- | --- |
| CONTROL Without SIgA | SIgA |  |  |  |  |  |  |  |  |  |
|  | IL-6 |  |  | 0.302 | 0.019 | 0.325 | 0.473 | 0.027 | 0.623 | 0.409 |
|  | IL-10 |  | 0.297 |  | 0.341 | 0.141 | 0.492 | 0.473 | 0.684 | 0.233 |
|  | IL-12 |  | 0.626 | -0.275 |  | 0.492 | 0.750 | 0.001 | 0.900 | 0.233 |
|  | IL-13 |  | 0.284 | 0.415 | -0.200 |  | 0.916 | 0.464 | 0.244 | 0.246 |
|  | IL-17A |  | 0.209 | -0.200 | -0.095 | 0.033 |  | 0.693 | 0.489 | 0.512 |
|  | IFN-α |  | 0.596 | -0.209 | 0.824 | -0.213 | 0.116 |  | 0.384 | 0.616 |
|  | IFN-β |  | 0.143 | 0.119 | 0.037 | 0.332 | 0.200 | 0.251 |  | 0.967 |
|  | IFN-γ |  | -0.240 | -0.341 | -0.341 | 0.332 | 0.191 | -0.147 | 0.013 |  |
|  |  | | | | | | | | | |
| CONTROL With SIgA | SIgA |  | 0.470 | 0.733 | 0.785 | 0.892 | 0.953 | 1.000 | 0.759 | 0.279 |
|  | IL-6 | 0.261 |  | 0.279 | 0.407 | 0.044 | 0.380 | 0.204 | 0.155 | 0.006 |
|  | IL-10 | 0.127 | 0.382 |  | 0.023 | 0.632 | 0.400 | 0.009 | 0.918 | 0.349 |
|  | IL-12 | 0.103 | 0.297 | 0.721 |  | 0.560 | 0.082 | 0.002 | 0.349 | 0.114 |
|  | IL-13 | 0.055 | -0.661 | -0.176 | 0.212 |  | 0.207 | 0.865 | 0.023 | 0.584 |
|  | IL-17A | 0.024 | -0.310 | 0.298 | 0.584 | 0.438 |  | 0.186 | 0.097 | 0.953 |
|  | IFN-α | -0.006 | 0.442 | 0.794 | 0.879 | -0.067 | 0.456 |  | 0.759 | 0.133 |
|  | IFN-β | -0.115 | 0.491 | 0.042 | -0.333 | -0.721 | -0.559 | -0.115 |  | 0.470 |
|  | IFN-γ | -0.382 | -0.818 | -0.333 | -0.539 | 0.200 | 0.024 | -0.515 | -0.261 |  |
|  |  |  |  |  |  |  |  |  |  |  |
| COVID-19 Without SIgA | SIgA |  |  |  |  |  |  |  |  |  |
|  | IL-6 |  |  | 0.426 | 0.795 | 0.910 | 0.306 | 0.426 | 0.209 | 0.001 |
|  | IL-10 |  | -0.242 |  | 0.726 | 0.639 | 0.353 | 0.723 | 0.448 | 0.289 |
|  | IL-12 |  | 0.080 | -0.107 |  | 0.001 | 0.024 | 0.004 | 0.938 | 0.733 |
|  | IL-13 |  | 0.036 | -0.143 | 0.812 |  | 0.197 | 0.043 | 0.439 | 0.569 |
|  | IL-17A |  | 0.308 | -0.280 | 0.628 | 0.382 |  | 0.209 | 0.325 | 0.505 |
|  | IFN-α |  | 0.242 | -0.110 | 0.749 | 0.575 | 0.374 |  | 0.949 | 0.176 |
|  | IFN-β |  | 0.374 | -0.231 | -0.025 | -0.234 | 0.297 | -0.022 |  | 0.373 |
|  | IFN-γ |  | 0.841 | -0.319 | 0.105 | 0.173 | 0.203 | 0.401 | 0.269 |  |
|  |  | | | | | | | | | |
| COVID-19 With SIgA | SIgA |  | 0.681 | 0.156 | 0.815 | 0.116 | 0.002 | 0.148 | 0.209 | 0.025 |
|  | IL-6 | -0.067 |  | 0.522 | 0.235 | 0.240 | 0.137 | 0.444 | 0.571 | 0.001 |
|  | IL-10 | 0.229 | 0.104 |  | 0.000 | 0.439 | 0.130 | 0.134 | 0.602 | 0.092 |
|  | IL-12 | 0.038 | 0.192 | 0.526 |  | 0.096 | 0.035 | 0.000 | 0.908 | 0.062 |
|  | IL-13 | 0.252 | 0.190 | 0.126 | 0.267 |  | 0.034 | 0.001 | 0.598 | 0.007 |
|  | IL-17A | 0.479 | -0.239 | 0.244 | 0.334 | 0.335 |  | 0.391 | 0.808 | 0.318 |
|  | IFN-α | 0.233 | 0.124 | 0.241 | 0.550 | 0.502 | 0.139 |  | 0.153 | 0.028 |
|  | IFN-β | 0.203 | 0.092 | -0.085 | -0.019 | 0.086 | -0.040 | 0.230 |  | 0.426 |
|  | IFN-γ | 0.353 | 0.492 | 0.270 | 0.298 | 0.419 | 0.162 | 0.348 | 0.129 |  |

Note: Pearson's r values are shown on the lower diagonal. p values are presented in the upper diagonal. Significant values were highlighted (r: green and p: blue). Significance level considered: p < 0.05.
